# CT- and MRI-Based Assessment of Body Composition and Pancreatic Fibrosis Reveals High Incidence of Clinically Significant Metabolic Changes That Affect the Quality of Life and Treatment Outcomes of Patients with Chronic Pancreatitis and Pancreatic Cancer

**DOI:** 10.3390/medicina55100649

**Published:** 2019-09-27

**Authors:** Edita Bieliuniene, Jens Brøndum Frøkjær, Alius Pockevicius, Jurate Kemesiene, Saulius Lukosevicius, Algidas Basevicius, Vaida Atstupenaite, Giedrius Barauskas, Povilas Ignatavicius, Antanas Gulbinas, Zilvinas Dambrauskas

**Affiliations:** 1 Department of Radiology, Lithuanian University of Health Sciences, Kaunas 50161, Lithuania; jurate.did@gmail.com (J.K.), saulius.lukosevicius@kaunoklinikos.lt (S.L.), algidas.basevicius@kaunoklinikos.lt (A.B.), avaida@gmail.com (V.A.); 2 Department of Radiology, Aalborg University Hospital, Aalborg 9000, Denmark; jebf@rn.dk; 3 Department of Clinical Medicine, Aalborg University, Aalborg 9000, Denmark; 4 Department of Veterinary Pathobiology, Lithuanian University of Health Sciences, Kaunas 44307, Lithuania; Alius.Pockevicius@lsmuni.lt; 5 Department of Surgery, Lithuanian University of Health Sciences, Kaunas 50161, Lithuania; Giedrius.Barauskas@kaunoklinikos.lt (G.B.); ignatavicius@gmail.com (P.I.); 6 School of Medicine, Nazarbayev University, Nur-Sultan 010000, Kazakhstan; gulbanta@gmail.com; 7 Institute for Digestive System Research, Lithuanian University of Health Sciences, Kaunas 44307, Lithuania; Zilvinas.Dambrauskas@lsmuni.lt

**Keywords:** pancreas, sarcopenia, CT, MRI, DXA, osteoporosis/osteopenia, quality of life

## Abstract

*Background and Objectives:* Both chronic pancreatitis (CP) and pancreatic ductal adenocarcinoma (PDAC) may lead to cachexia, sarcopenia, and osteoporosis due to different mechanisms. Neither patient gender, age, nor body weight are good predictors of these metabolic changes having a significant negative impact on the quality of life (QOL) and treatment outcomes. The aim of this study was to evaluate radiological changes in body composition and to compare them with manifestations of exocrine and endocrine pancreatic insufficiency, body mass, and QOL among patients with CP and PDAC. *Materials and Methods:* Prospectively collected data of 100 patients with diagnosed CP or PDAC were used for analysis. All patients underwent dual-energy X-ray absorptiometry (DXA), computed tomography (CT), and magnetic resonance imaging (MRI). The European Organization for Research and Treatment of Cancer Quality of Life Questionnaire-C30 (EORTC QLQ-C30) was used to assess QOL. Diabetes and changes in fecal elastase-1 were also assessed. *Results:* There was no significant difference in skeletal muscle mass (SMM) among patients with CP and PDAC (*p* = 0.85). Significantly more underweight patients had low SMM (*p* = 0.002). Patients with CP had more pronounced pancreatic fibrosis (PF) (*p* < 0.001). Data showed a significant relationship between a high degree of PF and occurrence of diabetes (*p* = 0.006) and low fecal elastase-1 levels (*p* = 0.013). A statistically significant lower QOL was determined in patients with PF ≥ 50% and in the CP group. *Conclusions:* Sarcopenia and osteoporosis/osteopenia are highly prevalent among patients with chronic pancreatitis and pancreatic cancer, and CT- and MRI-based assessment of body composition and pancreatic fibrosis could be a potentially useful tool for routine detection of these significant metabolic changes.

## 1. Introduction

Both chronic pancreatitis (CP) and pancreatic ductal adenocarcinoma (PDAC) may lead to cachexia, sarcopenia, and osteopenia due to different mechanisms. These changes might impair quality of life and/or limit treatment options and their effectiveness.

Pancreatic fibrosis develops in the course of CP. Characteristic features of advanced CP are destruction of acinar and islet cells, increased number of stromal cells, and prominent fibrosis [1]. Whatever the etiology of CP is, pancreatic atrophy with replacement of the glandular elements by fat and fibrous tissue appears to be a typical response to injury. Based on the known pathological changes, it is expected that atrophy of the pancreatic tissue would be characteristic for CP [2]. Clinical manifestations of the replacement of glandular elements and pancreatic fibrosis (PF), closely related to clinical symptoms of CP, include exocrine pancreatic insufficiency (steatorrhea) and diabetes mellitus. It leads to changes that affect a patient’s quality of life with such physical symptoms as diarrhea, nausea and vomiting, unexplained weight loss, excessive thirst and fatigue, disabling abdominal pain, and frailty, as well as psychological disorders such as depression and depression-related symptoms [3]. Early detection and quantification of fibrosis could potentially offer a possibility for early treatment, resulting in the improvement of clinical outcomes and maintenance of pancreatic function [4,5]. Fibrosis is very fundamental in the development of CP, and it usually develops at an early point when other imaging findings are not very pronounced. MRI could potentially be a sufficient biomarker for the assessment of pancreatic fibrosis and has been validated against histology [6].

Weight loss in pancreatic cancer can result from reduced food intake, abnormal metabolism, or most commonly, a variable combination of the two. Moreover, pancreatic fibrosis and metabolic changes develop with the advancement of PDAC [7,8]. Loss of adipose tissue (reflecting a negative energy balance) and reduction of skeletal muscle (reflecting a negative nitrogen balance) may or may not develop synchronously. Cachexia is associated with a lower tolerance for chemotherapy, which limits the total dose that can be delivered, the number of symptomatic responses, and any survival advantage that might be accrued [9,10,11].

Since both CT- and MRI-based approaches are used in the diagnosis of pancreatic disease, both modalities have also been extensively tested for the radiological assessment of body composition and metabolic changes in non-malignant and, more recently, malignant disease [5,9].

Metabolic changes (loss of overall weight and fat, sarcopenia, and osteopenia) could affect patient quality of life and treatment outcomes and thus mandate specific management.

The hypothesis of this study was that metabolic changes, detected by CT and MRI, and laboratory-diagnosed pancreatic exocrine and endocrine insufficiency correlate with pancreatic fibrosis and quality of life in CP and PDAC. To test the hypothesis, we sought to correlate radiological features with body composition (sarcopenia and loss of bone mass) changes, manifestations of exocrine or endocrine insufficiency, quality of life, and pancreatic fibrosis among patients with CP and PDAC.

## 2. Methods and Materials

### 2.1. Patients

We prospectively collected data of 104 patients with CP (*n* = 63) and PDAC (*n* = 37). Fifty-two of them with cancer (stage of the tumor was from T1 to T3 with no distant metastasis) and complicated CP underwent surgery. All patients underwent preoperative abdominal CT and MRI scans, and the final diagnoses were consequently confirmed postoperatively by histological examination of the surgical specimens. The stool samples for fecal elastase-1 estimation were collected at least 2 days before the operation. Other patients underwent the same examinations during their last inpatient stay. This was a consecutive group of patients treated in the Departments of Gastroenterology and Surgery, at the Hospital of the Lithuanian University of Health Sciences in the period from October 2016 to May 2018.

Four patients were excluded from the final analysis because it was not possible to measure and calculate the degree of sarcopenia due to the poor quality of CT images and patient refusal to repeat the scanning. There were 66 males and 34 females, with an average age of 55 years (range 22–89 years) and an average body mass index (BMI) of 24 kg/m^2^ (range 14–44) enrolled in the study. Body composition and pancreatic fibrosis variables of the patients are shown in Table 1.

Dual-energy X-ray absorptiometry (DXA), MRI, and CT imaging were performed in all cases before the surgical treatment and during the last inpatient stay for those who hadn’t been operated on.

Statement of Ethics: The prospective study was approved by the Kaunas Regional Biomedical Research Ethics Committee (Protocol No. BE-10-11, 20-07-2016).

#### 2.1.1. Quality of Life

During the last inpatient stay, all patients filled in the same questionnaire for quality of life assessment. We applied the European Organization for Research and Treatment of Cancer Quality of Life Questionnaire-C30 (EORTC QLQ-C30), which has been successfully used in other chronic pancreatitis and pancreatic cancer studies [12]. For the analysis, we used the following scales: Global health status (QL2), Physical functioning (PF2), Fatigue (FA), Pain (PA), Appetite loss (AP) and Diarrhoea (DI).

#### 2.1.2. Assessment of Pancreatic Volume

All CT examinations were performed with a 64-slice CT tomography unit GE Light Speed Pro (GE Healthcare, Milwaukee, WI, USA) with and without intravenous injection of 100 mL water-soluble iodine contrast medium (270–320 mg/mL) in portovenous phase. The slice thickness was 2.5 mm. All images were analyzed at a window level of 40 Hounsfield units (HU) and window width of 300 HU.

The pancreas was identified by using typical landmarks (splenic vein and superior mesenteric artery). Using the hand-outlined pancreas and surface toll in the Vitrea workstation (Vitrea, Vital Images, Canon Group Company, Minnetonka, Minnesota, USA), we measured the total pancreatic volume.

Pancreatic volume was measured using the summation-of-areas method, in which the outer margin of the pancreas was manually drawn as a cutting line to remove all surrounding structures (tumor volume, cyst volume, dilated ducts, etc. were excluded). This was repeated for every single slice containing pancreatic tissue. Total pancreatic volume (mL) was computed by summing up the volume from each slice (area from each slice × 2.5 m = volume) that included a piece of pancreatic tissue. The measurement of pancreatic volume was carried out by a single investigator.

### 2.2. Assessment of Skeletal Muscle Mass

**Figure 1:**
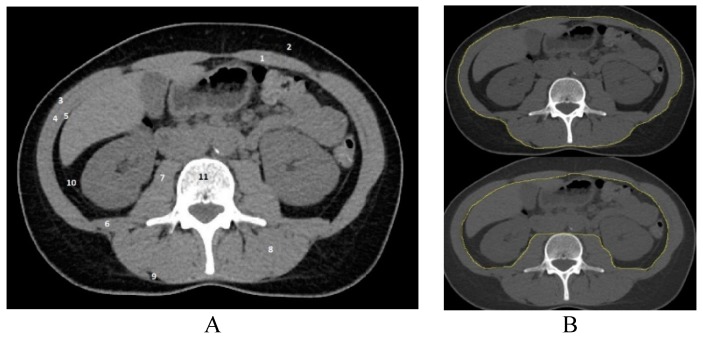
Axial CT image at the level of the L3 vertebra (**A**). Structures that are segmented for the assessment of sarcopenia: 1—m. rectus abdominis, 2—subcutaneous adipose tissue (SAT), 3—m. obliquus externus abdominis, 4—m. obliquus externus abdominis, 5—m. obliquus internus abdominis, 6—m. quadratus lumborum, 7—m. psoas major, 8—m. erector spinae, 9—intramuscular adipose tissue (IAT), 10—visceral adipose tissue (VAT), and 11—body of L3 vertebra. Axial CT image at the level of the L3 vertebra delineated with Image J “freehands selection” tool outer and inner abdominal musculature (**B**).

A single axial CT image at the level of the third lumbar vertebra was assessed to measure the cross-sectional areas of skeletal muscle (m. psoas major, m. erector spinae, m. quadratus lumborum, m. obliquus externus abdominis, m. obliquus internus abdominis, m. transversus abdominis, and m. rectus abdominis) (Figure 1). The tissue cross-sectional area at this level correlates well with total body skeletal muscle distribution [13]. Images were analyzed using NIH ImageJ (1.50i) software (Wayne Rasband, National Institutes of Health, Bethesda, Maryland, USA), which allows quantification of the tissue composition by using Hounsfield units (HU). The threshold for skeletal muscle was 150 to −29 HU. The area of skeletal muscle (SMA) was normalized for the height of the patient and the lumbar skeletal muscle index (L3 SMI) was calculated (cm^2^/m^2^) as SMA (cm^2^)/height (m^2^) [14].

Based on skeletal muscle cut-off values, defined from a large study in a healthy population, sarcopenia in patients was determined when the SMI was lower than 34.4 cm^2^/m^2^ for females and lower than 45.4 cm^2^/m^2^ for males [15].

The cut-off values associated with health or disease for the CT-defined abdominal adipose depots have not been determined, rendering this information somewhat arbitrary and clinically uninterpretable at this time [14].

### 2.3. MRI Examination

All MRI examinations were performed by means of a 1.5 T Siemens Magnetom Area (Siemens Healthcare GmbH, Erlangen, Germany) using a torso multi-coil array. Included image sequences were as follows: (1) axial and coronal T2-weighted images (slice thickness, 5 mm; repetition time (TR): 13.800 milliseconds; echo time (TE): 89 milliseconds; flip angle, 90 degrees; matrix size, 320 × 224; and field of view (FOV), 324 mm); (2) Diffusion-weighted imaging (DWI) with multiple *b*-values 50, 400, and 800 s/mm^2^ (axial echo planar imaging, slice thickness, 5 mm; TR, 6000 milliseconds; TE, 52.2 milliseconds; matrix size, 128 × 128; FOV, 400 mm) and apparent diffusion coefficient (ADC) maps, which were calculated automatically for each section by the imager software; and (3) axial T1-weighted images (slice thickness, 3 mm; TR, 7.14 milliseconds; TE, 2.38 milliseconds; flip angle, 10 degrees; and FOV, 380 mm).

Pancreatic tissue was identified on the T1, T2, and DWI sequences. Patients with chronic pancreatitis were examined during their inpatient stay, 1–3 months before surgery, and patients with pancreatic cancer were examined on the day before surgery. The largest possible region of interest (ROI) was used for each patient and was repeated three times with different ROIs. The ROI was kept at a maximum of 200 mm^2^ and a minimum of 50 mm^2^. The positions of the ROIs were guided by the axial T2-weighted images, and ducts, cysts, and artefacts were avoided.

In patients who underwent pancreas resection (*n* = 52), ADC and T1 signal intensity (SI) was measured at the anticipated resection margin selected under the surgeon’s supervision. CT scan images were evaluated and compared with MRI scan images for the identification of tumor margins. Measurements were performed approximately 1–3 cm from the margins of the tumor, in complex with the pancreas resection margin. The boundaries of each tumor were identified by means of contrast-enhanced CT scan and MR T1 and DWI sequences. The average of the three measurements was accepted as the final ADC value of the segment. All ADC values were measured directly from the ADC map data on an independent workstation. Since fibrotic pancreatic tissue is known to have a more hypo-intense signal intensity (SI) in fat-suppressed T1-weighted imaging, quantitative T1 SI values were measured on the T1 Dixon water-only images in the same location as the ADC [6].

For the remaining patients, who had not undergone histological verification, ADC values were measured in the pancreas head/body projection (since histologically tested patients’ measurements were performed in a similar location).

All of the radiological examinations were evaluated by a radiologist with at least 10 years of experience who was blinded to the histology results.

### 2.4. Assessment of Bone Density

Bone density was determined by using dual-energy X-ray absorption (DXA) (QDR-1000, Hologic Instruments, Waltham, MA, USA) at the standard measurement sites in the lumbar spine and femur. Results are presented as *T* scores and *Z* scores: normal bone density (*T* score > −1), osteopenia (*T* score from −1 to −2.5), and osteoporosis (*T* score < −2.5).

### 2.5. Pancreatic Exocrine Function Testing

Pancreatic exocrine function was tested on the day before surgery using the ScheBo^®^ Pancreatic Elastase Stool test (ScheBo, Mannheim, Germany). Fecal elastase-1 test results exceeding 200–500 µg/g were considered normal, whereas results less than 200 µg/g were considered moderate and less than 100 µg/g indicated strong pancreatic exocrine insufficiency [16].

### 2.6. Statistical Analysis

Statistical analysis was performed using SPSS 22.0 (SPSS, Inc., Chicago, IL, USA) software. Quantitative parameters between independent groups were compared using independent sample *T* tests, Pearson and Spearman correlations, and Mann–Whitney–*U* tests. A *p*-value of less than 0.05 was considered statistically significant.

## 3. Results

### 3.1. Sarcopenia and Low Bone Density in Patients with Chronic Pancreatitis and Pancreatic Cancer

Analysis identified 34 (34%) patients with sarcopenia. Neither patient’s gender, age, nor mean body weight were statistically significantly related to sarcopenia. There were significantly more underweight patients (with BMI < 18.5 kg/m^2^) who had sarcopenia (*p* = 0.002), but 80% of sarcopenic patients had a normal BMI or were overweight (Table 2). There was no significant difference in sarcopenia status among the patients with CP and PDAC (*p* = 0.85). The presence of osteopenia/osteoporosis predicts the presence of sarcopenia (*p* = 0.02) (Table 2).

**Figure 2:**
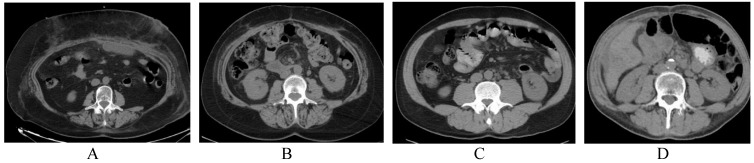
Illustrative examples of mismatch between BMI and sarcopenia in CP and PDAC patients. Images of sarcopenic female patient with PDAC at the level of L3 (BMI = 19 kg/m^2^ and lumbar skeletal muscle index (L3 SMI) = 37 cm^2^/m^2^) (**A**) and sarcopenic female with PanCa at the level of L3 (BMI = 44 kg/m^2^ and L3 SMI = 34 cm^2^/m^2^) (**B**). Images of non-sarcopenic female patient with CP at the level of L3 (BMI = 18 kg/m^2^ and L3 SMI = 40 cm^2^/m^2^) (**C**) and non-sarcopenic male patient with CP at the level of L3 (BMI = 29 kg/m^2^ and L3 SMI = 55 cm^2^/m^2^ (**D**).

In the PDAC group, 13 out of 34 (38%) patients were sarcopenic, with a mean L3 SMI of 46.06 ± 9.3 cm^2^/m^2^ for men and 40.43 ± 7.07 cm^2^/m^2^ for women. The mean BMI in the PADC group was 25.54 ± 5.2 kg/m^2^, and the presence of sarcopenia did not always correlate with BMI values, as illustrated in Figure 2A,B. Sarcopenia was diagnosed in 1 out of 13 (8%) patients with decreased BMI (<18.5 kg/m^2^), in 7 out of 13 (54%) patients with normal BMI (18.5–24.9 kg/m^2^), and in 3 out of 13 (23%) patients with increased BMI (25–29.9 kg/m^2^). Two out of 5 (40%) obese patients (BMI > 30 kg/m^2^) also had sarcopenia.

In the CP group, 21 out of 34 patients (62%) were sarcopenic, with a mean L3 SMI of 49.60 ± 7.5 cm^2^/m^2^ for men and 47.00 ± 8.6 cm^2^/m for women. The mean value of BMI in the CP group was 24.08 ± 4.5 kg/m^2^. CT images of two non-sarcopenic patients with CP and different BMI values are shown in Figure 2C,D. Sarcopenia was diagnosed in 6 out of 21 (29%) patients with decreased BMI (<18.5 kg/m^2^), in 11 out of 21 (52%) patients with normal BMI (18.5–24.9 kg/m^2^), and in 3 out of 21 (14%) patients with increased BMI (25–29.9 kg/m^2^), again illustrating that body weight alone is a poor indicator of the significant changes in body composition and metabolism.

An in-depth analysis of the association between sarcopenia and pancreatic volume, exocrine and endocrine pancreatic insufficiency, and bone density revealed that only abnormal bone mass (osteopenia and/or osteoporosis) was significantly associated with sarcopenia (*p* = 0.02) (Table 2).

In a further analysis, we explored the association between pancreatic fibrosis (PF) and metabolic changes.

Based on ADC and T1 measurements of the pancreas and using cut-off values of ADC < 1.316 × 10^−3^ mm^2^/s and T1 < 170 units for predicting ≥50% PF as defined in our previous study [6], we divided our patients into two groups: low-grade (*n* = 39) and high-grade (*n* = 61) PF.

The patients with CP had a higher degree of PF (*p* < 0.001). There was no direct association between the severity of PF and sarcopenia (*p* = 0.45) (Table 3). However, the data showed a significant relationship between the higher degree of PF and occurrence of diabetes (*p* = 0.006), as well as low fecal elastase-1 levels (*p* = 0.013) (Table 3).

### 3.2. Symptoms Influencing Quality of Life in Patients with Severe Pancreatic Fibrosis and CP

Sarcopenia, diabetes, and bone density changes did not affect quality of life. However, a statistically significant lower quality of life in the QL2 scale was determined in patients with PF ≥ 50%, with 50.00 (33.33) points as compared to 66.66 (25.00) points in patients with PF < 50% (*p* < 0.001).

Statistically significant differences were also obtained in the Physical functioning (PF2), Fatigue (FA), Pain (PA), and Appetite loss (AP) scales. In the diarrhea (DI) scale, there were no statistically significant difference.

The PF2 scale resulted in 73.33 (33.33) points in the PF ≥ 50% group vs. 80.00 (33.33) points in the PF < 50% group (*p* = 0.005).

**Figure 3:**
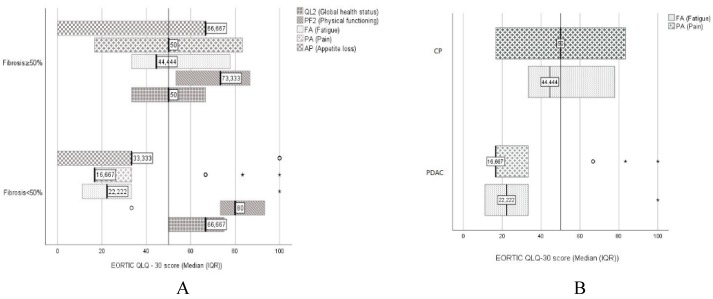
Different scales of the quality of life results in patients with a low and high degree of PF (**A**) and in patients with chronic pancreatitis and pancreatic cancer (**B**). A high score represents a high level of symptomatology/problems in the FA, PA, and AP scales. In the PF2 scale, a high score represents a high/healthy level of function, and in the QL2 scale, a high quality of life.

The PA scale showed a result of 50 (66.67 IQR) points in the PF ≥ 50% group vs. 16.66 (16.67 IQR) points in the PF < 50% group (*p* = 0.003). The AP scale was 66.66 (66.67 IQR) points in the PF ≥ 50% group vs. 33.33 (33.33 IQR) points in the PF < 50% group (*p* = 0.004) (Figure 3A).

In patients with low fecal elastase-1 values, there was a statistically significant difference observed in the FA scale: 22.22 (44.44 IQR) points in fecal elastase-1 > 200 µg/L vs. 44.44 (33.33 IQR) points in fecal elastase-1 ≤ 200 µg/L (*p* = 0.009).

There was no statistically significant difference in global health status between groups. The FA scale results were significantly different in the CP group 44.44 (44.44 IQR) points vs. 22.22 (22.22 IQR) points in the PDAC group (*p* < 0.001).

There was a significant difference in the pain scale between groups: 50 (66.67 IQR) points in the CP group vs. 16.66 (16.67 IQR) points in the PDAC group (*p* < 0.001). CP patients had a lower quality of life as compared with PDAC patients: 50.00 (33.33 IQR) points in the CP group vs. 66.66 (16.67 IQR) points in the PDAC group; however, the difference was non-significant (*p* = 0.77) (Figure 3B).

## 4. Discussion

Metabolic changes during the disease strongly affected patient quality of life and treatment outcomes; therefore, we have evaluated them by using objective radiological (for sarcopenia and bone density changes) and laboratory (for diabetes and fecal elastase-1 values) tests. In this study, we included patients with pancreatic cancer and chronic pancreatitis who underwent elective CT and MRI studies for the evaluation of their principal disease and treatment planning; however, we focused on the value of these radiological modalities in the assessment of the metabolic changes.

Our data shows that loss of skeletal muscle and bone mass is highly prevalent among patients with CP and PDAC, independent of the type of disease. Analysis of the relationship between sarcopenia and exocrine as well as endocrine pancreatic insufficiency (PEI) revealed that only abnormal bone mass (osteopenia and/or osteoporosis) was significantly associated with sarcopenia. Similarly, in the study of 132 patients with pancreatic diseases, Shintakuya et al. demonstrated a clear relationship between sarcopenia and PEI.

Sarcopenia is associated with many clinical conditions, such as cancer, diabetes, acquired immune deficiency syndrome, burns, chronic obstructive pulmonary disease, chronic heart failure, chronic renal failure, chronic pancreatitis, rheumatoid arthritis, and sepsis [17,18,19]. Sarcopenia is defined as depletion of skeletal muscle mass with a risk of adverse outcomes, such as physical disability and poor quality of life. The process starts around the age of 40 and progresses at a rate of 8% loss of muscle tissue per decade until the age of 70, when muscle loss accelerates to 15% per decade [20]. Similarly in our study, the mean age of patients with sarcopenia was 58, 32 ± 16.46 years. A significantly larger number of cases of sarcopenia were observed in patients with chronic pancreatitis (62%), suggesting that sarcopenia was more frequent in patients with chronic illnesses as compared to cancer patients.

Many important features of body composition could be overlooked when assessing only overall body mass, BMI and/or changes in these parameters (i.e., percentage of weight loss/gain) [21]. Imaging-based examination of body composition is highly differentiated, allowing separate discrimination of many facets, including low bone mass/density (osteopenia/osteoporosis), excess fat (obesity), low muscle mass (sarcopenia), and the combination of excess fat with low muscle mass (sarcopenic obesity) [21].

A BMI < 18.5 kg/m^2^ is considered to represent an individual at serious risk of undernutrition by many authors [4]. In the present study, only 10% of individuals at baseline fulfilled this criterion. Given the prevalence of overweight/obesity (40%), it would seem unlikely that, even in the presence of ongoing weight loss, the majority would reach this boundary at or near the time of death.

Analysis of body composition revealed that sarcopenia is highly prevalent in patients with pancreatic cancer and chronic pancreatitis (34% in our group of patients) and may be present in patients with any BMI value. In our study, 80% of patients had sarcopenia and were of normal weight or overweight/obesity, and only 8% of patients had sarcopenia and were underweight, clearly suggesting the limitations of BMI; a more detailed evaluation of body composition clearly revealed wasting of the lean tissues in the majority of patients.

Being below or well below benchmark levels of muscularity is known to be associated with higher mortality and functional disability [5]. In the current literature, it is becoming increasingly evident that concurrent sarcopenia and high fat mass is the worst-case scenario [10,22,23], and this was clearly shown in the study by Tan et al., where sarcopenic overweight/obese patients had the worst overall prognosis, even compared with patients who were sarcopenic and had a lower body weight [10]. In our research, 24% of patients were overweight/obese and had sarcopenia.

Another important message from the literature is that sarcopenia is a strong predictor of the occurrence of pancreatic fistula and patient survival after pancreatoduodenectomy. These observations call for reconditioning of sarcopenic patients before pancreatoduodenectomy [24].

Ratnayake et al. performed a meta-analysis including 3608 patients from thirteen studies. There was a significant increase in the mean duration of postoperative hospital stay, but there was no difference in postoperative outcomes, including clinically relevant postoperative pancreatic fistula, delayed gastric emptying, postoperative bile leak, surgical site infection, significant morbidity, and overall morbidity [25].

Guinoti et al. reviewed the best contemporary literature and worked to develop a position paper to provide evidence supporting the integration of appropriate nutritional support into the overall management of patients undergoing pancreatic resection. In their paper, the authors proved that malnutrition is a recognized risk factor for surgery-related complications; therefore, the measurement of nutritional status should be a part of routine preoperative assessment [26]. In addition to patient’s nutritional status, weight loss and body mass index measurement of sarcopenia and sarcopenic obesity should also be considered in the preoperative evaluation because they are strong predictors of poor short-term and long-term outcomes [26].

The pancreas has a complex histology and is characterized by a combination of endocrine and exocrine cells. The pancreas undergoes various pathological changes with ageing, characterized by increased fatty replacement, fibrosis, lymphoplasmacytic infiltration, amyloid deposition, and decreased weight as well as development of intra-epithelial neoplastic changes. Pancreatic fibrosis, as well as steatosis, can develop in older people as age-related changes [23]. Lymphoplasmacytic infiltration and fibrosis in the interlobular septa are often seen in the autopsied elderly. This change is named chronic interstitial pancreatitis, and post-mortem studies of patients with uremia, chronic inflammatory bowel diseases, cachexia, malnutrition, or extensive exhaustion for any reason demonstrate a high frequency of mild inflammatory changes in the pancreas [23]. Decreased secretion of digestive enzymes due to the tumor and/or pancreatic fibrosis in CP results in loss of the ability to absorb macronutrients adequately. Deficiencies of essential nutrients increase health risks and produce serious symptoms [22,27,28].

Our data show that PF develops much earlier in patients with CP (mean age of 59 ± 2.3 years in the low-grade fibrosis group and 53 ± 1.8 years in the high-grade fibrosis group) and comes with severe complications such as sarcopenia, osteoporosis, and diabetes.

After analyzing the low-grade and high-grade PF groups, we found that patients with CP had a higher degree of PF (the highest degrees of PF were established for 80% of patients with CP). Patients with higher degrees of PF have a tendency to develop DM (34%) and exocrine pancreas insufficiency (80% had low fecal elastase-1 levels). In CP and pancreatic cancer patients, fecal elastase-1 values were significantly different (157.17 ± 184.07 µg/L vs. 310.38 ± 196.87 µg/L, respectively).

Osteoporosis and sarcopenia are two chronic diseases representing a major clinical problem, with increasing prevalence in the elderly. The combination of these diseases has a non-negligible impact on the quality of life and survival rate due to the increased possibility of falls, fractures, and frailty [29]. In our study, these changes were found in much younger patients. The mean age of patients with osteoporosis/osteopenia was 57 years. Sarcopenia was present in 34% of patients, and 65% of them were diagnosed with low bone density.

An article on nutritional therapy, as well as our study, states that chronic pancreatitis is associated with osteoporosis, sarcopenia, poor quality of life, and increased mortality [30].

Another study by Duggan et al. revealed that osteoporosis/osteopenia afflicts two-thirds of patients as a consequence of poor dietary intake of calcium and vitamin D and low physical activity as well as chronic low-grade inflammation. Moreover, bone metabolism studies showed increased bone resorption in patients with chronic pancreatitis [31]. Our study is in accordance, revealing significant correlation of sarcopenia and osteopenia/osteoporosis (65% of sarcopenic patients), as well as sarcopenia and decreased quality of life in patients with chronic pancreatitis.

Numerous studies have shown that chronic pancreatitis is associated with deterioration in QOL. Severity of pain, pain-related disability/unemployment, structural pancreatic changes, chronic diarrhea, low body weight, disease duration, and other factors adversely affect QOL [32,33,34].

In our study, fatigue and pain scales were significantly different in the CP group as compared to the PDAC group. Moreover, patients with a higher degree of PF had a worse quality of life. There was a statistically significant lower quality of life in the QL2 scale in patients with PF ≥ 50% as compared to patients with PF < 50%. Statistically significant differences were also obtained in physical functioning, pain, and appetite loss scales in these groups. In patients with low fecal elastase-1 values, a statistically significant difference was observed only in the fatigue scale.

Evaluating patients with pancreatic diseases and according to the results of his studies, Pezzilli recommends that quality of life be routinely assessed [35].

## 5. Conclusions

Sarcopenia and osteopenia are related and highly prevalent among chronic pancreatitis and pancreatic cancer patients, independent of the main disease, body weight, and/or pancreatic fibrosis. Patients with CP have a higher degree of PF, which is related to the occurrence of diabetes and low fecal elastase-1 levels, representing pancreatic exocrine insufficiency. Fecal elastase-1 as well as abnormal bone density and the presence of diabetes has a moderate overall accuracy for predicting exocrine insufficiency. The use of MRI for evaluation of PF and CT for body composition opens up new possibilities for the diagnosis and management of metabolic disorders among patients with chronic pancreatitis and pancreatic cancer and could potentially be a useful tool for routine assessment and treatment planning. DXA should be used for the evaluation of bone density status, especially in sarcopenic patients with pancreatic disease, as the presence of osteopenia could predict the presence of sarcopenia

### Study Limitations

Our study has a number of limitations. Firstly, the study population is comprised of a relatively small number of patients from a single institution. Secondly, both pancreatic cancer and chronic pancreatitis patient data were analyzed in subgroups and as a mixed population and the majority of patients had other illnesses, which might have affected the results. However, it was not possible to take into consideration all of the comorbidities. Thirdly, only half of the patients had histological tissue examination (only those who underwent surgery); in other cases, diagnoses were made by using CT or MRI combined with clinical examination. In addition, the reporting radiologists were aware of the clinical conditions and symptoms of patients and were expecting to find a lesion in the pancreas. Moreover, it is known that sarcopenia develops during ageing. In our study, we included only patients with diagnosed pancreatic pathology, and no healthy controls were examined. Therefore, we cannot clearly rule out ageing as a factor in the development of sarcopenia.

Despite these limitations, we can suggest that further work could be turned into a multi-centric study, including a greater amount of clinical cases and adding healthy controls, to improve the statistical results and clinical value.

## Figures and Tables

**Table 1:** Overview of body composition and pancreatic fibrosis variables divided by sex.

	Male (*n* = 66)	Female (*n* = 34)	*p* Value (chi square)
Weight (kg ± SD)	74.86 ± 12.70	68.21 ± 11.42	0.627
Mean age (years ± SD)	53.38 ± 14.49	58.88 ± 14.01	
BMI (kg/m^2^) mean ± SD	23.80 ± 3.88	24.08 ± 4.47	0.217
Patients (no):			
Underweight *	4 (6%)	4 (12%)	
Normal weight *	41 (62%)	15 (44%)	
Overweight and obesity *	21 (32%)	15 (44%)	
Sarcopenia (no)	25 (38%)	9 (26%)	0.254
Abnormal bone density (no) (osteopenia/osteoporosis)	33 (50%)	16 (47%)	0.694
Pancreatic fibrosis (%)	51.20 ± 32.60	49.81 ± 31.66	0.381
Mean (± SD)	(*n* = 30)	(*n* = 22)	

* Underweight: < 18.5; normal weight: 18.5–24.9; overweight: 25–29.9; obesity: ≥ 30. BMI: body mass index.

**Table 2:** Patients with normal skeletal muscle mass compared to sarcopenic patients. Relation between sarcopenia and pancreatic volume, exocrine and endocrine pancreatic insufficiency, and other metabolism disorders.

	Normal Muscle Mass (*n* = 66)	Sarcopenia (*n* = 34)	*p* Value
			(chi square)
Mean weight (kg)	74.64 ± 14.02	68.21 ± 11.42	0.28
BMI (kg/m^2^) mean ± SD	25.14 ± 4.33	22.03 ± 4.07	0.002
Patients (no):			
Underweight *	1 (2%)	7 (21%)	
Normal weight *	37 (56%)	19 (56%)	
Overweight and obesity *	28 (42%)	8 (24%)	
Gender (female/male)	25/41	9/25	0.25
Mean age (years ± SD)	53.67 ± 13.23	58.32 ± 16.46	0.391
Cancer (no)	24 (36%)	13 (38%)	
Chronic pancreatitis (no)	42 (64%)	21 (62%)	0.85
Abnormal bone density (no) (osteopenia/osteoporosis)	27 (41%)	22 (65%)	0.02
Pancreas volume (mL) mean (SD)	43.21 (27.65)	39.82 (19.86)	0.37
Diabetes mellitus (DM) (no)	19 (29%)	7 (21%)	0.52
Fecal elastase-1 < 200 µg/g (no)	40 (61%)	21 (62%)	0.91
Fecal elastase-1 Median	130.50 (183.171)	111.00 (188.593)	0.375
(interquartile range(IQR))			

* Underweight: <18.5; normal weight: 18.5–24.9; overweight: 25–29.9; obesity: ≥30.

**Table 3:** Association of PF with sarcopenia and underlying disease.

	<50% Fibrosis	≥50% Fibrosis	*p* Value (chi square)
Number of patients	39	61	
Age (years ± SD)	59 ± 2.3	53 ± 1.8	
Osteopenia/osteoporosis (no)	18 (46%)	31 (51%)	0.649
Sarcopenia yes no	15 (38%) 24 (62%)	19 (31%) 42 (69%)	0.45
Pathology Ca * CP	25 (64%) 14 (36%)	12 (20%) 49 (80%)	<0.001
Pancreas volume (mL) Mean (SD)	51.82 (25.20)	35.82 (23.35)	0.422
DM (no)	5 (13%)	21 (34%)	0.006
Fecal elastase-1 < 200 µg/g (no)	12 (31%)	49 (80%)	<0.001
Fecal elastase-1 median (IQR)	445.00 (179.48)	123.00 (184.07)	0.013

* Ca—pancreas and p. Vateri cancer.
